# Hemophagocytic Lymphohistiocytosis in a Patient with Hodgkin Lymphoma, HIV, and Epstein-Barr Virus

**DOI:** 10.7759/cureus.38382

**Published:** 2023-05-01

**Authors:** Faiza Javed, Mahmoud Amr, Ahmed H Abdelfattah

**Affiliations:** 1 Hospital Medicine, University of Kentucky, Lexington, USA

**Keywords:** concurrent chemo radiotehrapy, hiv aids, hodgkin lymphona, hemophagocytic lymphohistiocytosis (hlh), human immunodeficiency virus (hiv) infection

## Abstract

Hemophagocytic lymphohistiocytosis (HLH) is an aggressive and life-threatening syndrome of excessive immune activation. It occurs in many underlying conditions and all age groups due to severe and uncontrolled inflammatory reactions, with the resultant overproduction of immune cells and cytokines. This leads to multi-organ damage (if not detected early and treated properly) with a mortality of more than 55%. We present a case of a 38-year-old male patient who presented with HLH with concurrent HIV/AIDS, and Epstein-Barr virus (EBV)-related Hodgkin lymphoma. We aim to emphasize the importance of considering HLH and cancer in patients with HIV/AIDS.

## Introduction

Hemophagocytic lymphohistiocytosis (HLH) is caused by excessive inflammation and overactivation of the immune system which leads to multi-organ damage. The state of immune overactivation is thought to be caused by the absence of normal downregulation by activated macrophages and lymphocytes [1]. This can be either primary HLH syndrome which is related to a genetic disorder or can be secondary HLH disease which is associated with immune activation in the setting of an underlying disease or disorder and can be from an infectious, malignancy, or rheumatologic disorder [2]. HLH is more prevalent in infants, however, it can present in all ages, with a slight predisposition to males in adult patients. The diagnosis of HLH can be challenging, and it depends on clinical, laboratory, and histopathologic findings. 

## Case presentation

A 38-year-old male, who was recently diagnosed with HIV/AIDS (last CD 4 count 71 cells/mm3 and a viral load of >300k copies) and had not been compliant with antiretroviral therapy, presented to the hospital for subjective fevers. He denied any cough, congestion, shortness of breath, abdominal pain, nausea, diarrhea, headache, neck stiffness, or vision changes. On arrival, he was febrile at 102°F; his heart rate was in the 150s range, and he had an oxygen saturation of 98% on room air. Physical examination showed a cachectic-looking male with tachycardia and normal respiratory, neurological, abdominal, and musculoskeletal examination. Initial laboratory workups showed pancytopenia, microcytic anemia, elevated liver enzymes, hypertriglyceridemia, and high inflammatory markers (C-reactive protein and ferritin) (Table 1).

**Table 1:** Patients' laboratory investigations pre and post treatment. U/L: units per liter. mg/L: milli gram per liter, ug/L: nanogram per liter

LABORATORY INVESTIGATIONS	PRE-TREATMENT	POST- TREATMENT	REFERENCE RANGE	UNITS
White blood cells	1.76	2.30	4.5-11	10*3/uL
Haemoglobin	8.1	9.2	13-16	mg/dL
Platelets	113	128	150-450	10*3/uL
Alanine transaminase (ALT)	195	78	4-36	U/L
Aspartate transaminase (AST)	63	22	8-33	U/L
C- reactive protein (CRP)	139	29	<10	mg/L
Procalcitonin	1.94	0.78	<0.1	ug/dL
Triglycerides	307	190	<150	mg/dL
Creatinine	0.45	0.45	0.7-1.3	mg/dL
Lactate dehydrogenase (LDH)	388	162	140-280	U/L

**Figure 1:**
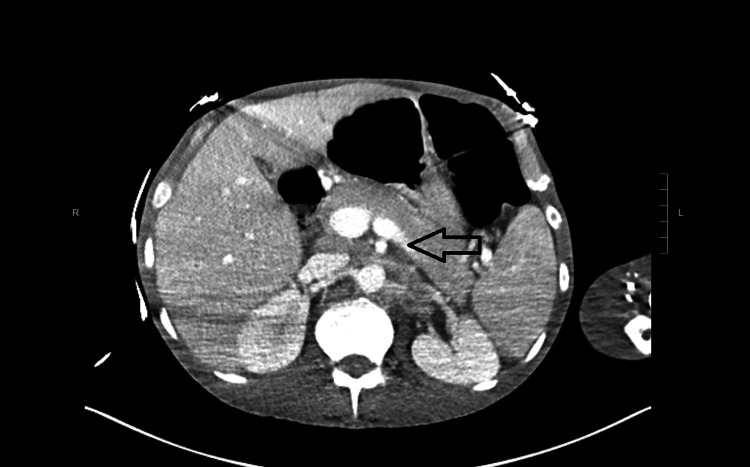
CT abdomen showing extensive retroperitoneal and pelvic adenopathy.

With high suspicion of sepsis, he was started on vancomycin, cefepime, and amphotericin empirically. CT of the abdomen and pelvis showed mild splenomegaly with extensive retroperitoneal and pelvic adenopathy (Figure 1).

**Figure 2:**
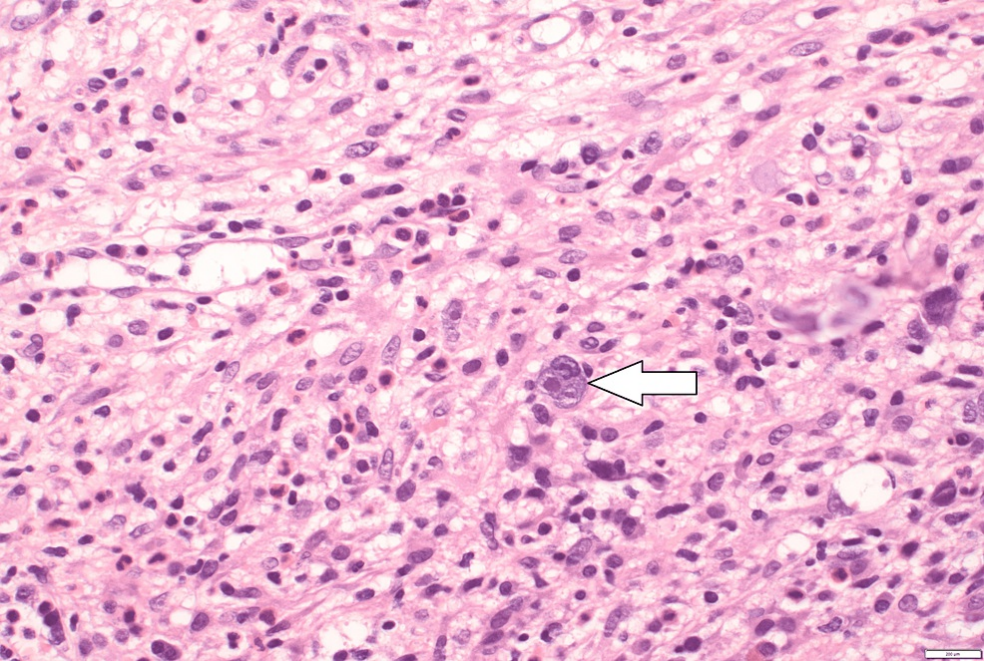
Bone marrow biopsy showing Reed Sternberg cells (arrow).

CT lungs showed mild ground glass opacities in the right upper lobe. Blood cultures, chlamydia, gonorrhea, methicillin-resistant Staphylococcus aureus (MRSA) PCR, syphilis/hepatitis screening, urine histoplasmosis antigen, Blastomyces antigen, toxoplasma antibody, cytomegalovirus (CMV) DNA, beta D-glucan and human leukocyte antigens (HLA) B5701 all came back negative. EBV came back positive with a viral load of 200K copies. Lymph node biopsy was offered to the patient, however, he refused to undergo it. He continued to be febrile despite starting antibiotics and the suspicion for HLH rose. Soluble interleukin-1 receptor level was elevated at 25,979 U/mL. At this point, the patient met 6/8 criteria based on the HLH-2004 scoring system, sufficient for HLH diagnosis. A bone marrow biopsy was performed, and antibiotics were discontinued. He was started on dexamethasone 16 mg per day as per HLH-94 protocol. The patient also agreed to start Biktarvy (bictegravir, emtricitabine, tenofovir alafenamide) for his HIV treatment. Bone marrow pathology had pancytopenia with extensive involvement by Hodgkin lymphoma cells and large inclusion-like nucleoli consistent with Reed-Sternberg cells. On immunohistochemistry, large atypical cells were positive for CD30, CD15, PAX5, and EBER and negative for CD20 and CD45 (Figure 2).

The patient was started on an AVD (doxorubicin, vinblastine, dacarbazine) chemotherapy regimen on days 1 and 15, along with rituximab, per the hematology team's recommendations. In the next few days, the patient’s clinical picture continued to improve; he became afebrile and all inflammatory markers started to trend downward (Figure 1). Additionally, the EBV viral load dropped to 1100 copies. He successfully completed the first cycle of chemotherapy with no complications and was discharged with a plan to return to the hospital for the second cycle. 

## Discussion

HLH, also known as hemophagocytic syndrome, is a rare life-threatening condition. Its incidence is around 1 in 100,000 which makes it underrecognized by clinicians [3]. 

HLH can occur as a primary hereditary immune disorder or it can be secondary to various conditions which include infections, malignancy, immunosuppression, post-allogeneic hematopoietic stem cell transplantation (allo-HSCT), and drug hypersensitivity [4]. The exact mechanisms causing the primary HLH remain uncertain; it could be due to genetic defects in some proteins and granzymes which play a significant role in the intracellular apoptosis process [5]. Both primary and secondary HLH is characterized by hypercytokinemia which can cause bone marrow suppression and damage to the vascular endothelium [4]. As mentioned before, malignancy and infection can precipitate HLH like lymphoma, EBV, and HIV infection like in our patient. Early diagnosis of HLH is crucial as it is an extremely aggressive condition. Diagnosis can be made by using the HLH-2004 criteria and HScore which have good diagnostic accuracy [6]. The HLH-2004 criteria are shown in Table 2. 

**Table 2:** The hemophagocytic lymphohistiocytosis (HLH) 2004 criteria. [7]

HLH- 2004 criteria [7]
Diagnosis of HLH requires one of the following criteria
Presence of molecular mutations* related to HLH
Or
Presence of five of the eight following criteria:
Fever (temperature ≥ 38.5°C)
Presence of splenomegaly
Cytopenia (affecting ≥ 2 cell lineages):
a. Hemoglobin <9 g/dl
b. Platelets <100 X 10^3/ml
c. Neutrophils <1 X 10^3/ml
Hypertriglyceridemia (265 mg/dL or more) and/or hypofibroginemia (less than 150 mg/dl)
Low or absent NK T-cell** activity
Elevated ferritin more than 500 ng/ml
Elevated soluble form of DC25 (α-chain of SIL-2Rα***
Histopathologic evidence of hemophagocytosis in bone marrow, spleen, liver or lymph node biopsy.
* PRF1, UNC13D, Munc18-2, Rab27a, STX11, SH2D1A, or BIRC4 ** Natural killer T-cell *** Soluble interleukin-2 receptor

The HScore uses a sum score of nine variables to calculate the likelihood of HLH. Each variable was given a number fluctuating between 18 and 64 points [3]. An H-score greater than 250 confers a 99% probability of HLH and a score of less than 90 confers a less than 1% probability of HLH. 

Our patient was diagnosed with secondary HLH in the setting of Hodgkin lymphoma, HIV, and EBV infection based on the HLH-2004 criteria. Immune overactivation was present in our patients with fever, fatigue, and elevated inflammatory markers. However, it was not entirely clear whether his presentation was solely related to Hodgkin lymphoma or associated with HIV and EBV.

Other differential diagnoses are possible. Immune reconstitution syndrome (IRIS) may present similarly, however, our patient presented with these symptoms and findings before starting any antiretroviral therapy. Another differential diagnosis is multicentric Castleman disease (MCD); however, in the presence of Hodgkin lymphoma, and the patient fulfilling the HLH-2004 criteria, the possibility of MCD is less likely.

HLH needs to be considered and recognized early. Its overlap with other infectious processes renders its diagnosis more challenging as many infections can present with fever, splenomegaly, and high ferritin levels [8]. Bone marrow biopsy is essential to diagnose HLH to detect hemophagocytosis and to exclude other causes of cytopenia [9]. 

The main treatment for secondary HLH is to treat the underlying cause with good supportive care, however, immunosuppression is often required. Dexamethasone is the first-line drug used in the immunosuppression of secondary HLH cases according to the HLH-94 treatment protocol [8]. In some cases, we may need to use etoposide and cyclosporine when dexamethasone fails to achieve the required response. Hematopoietic stem cell transplant may be an option for the definitive treatment of HLH in selected patients. 

The mortality rate remains high in patients with HLH. In one case series that included 162 patients, the mortality rate reached 43% with treatment, with a higher mortality rate observed among patients with hematologic malignancy [10,11]. It is important to note that early diagnosis and prompt treatment are critical in improving the chances of survival for patients with HLH, as the mortality rate can reach 50%-70% if left untreated [7]. 

## Conclusions

In conclusion, this case highlights the diagnostic challenge and clinical complexity in patients presenting with HLH. The identification of underlying etiologies is crucial for the proper management and treatment of HLH. In this case, our patient presented with fever, and was later found to have HIV with EBV in addition to the diagnosis of HL. This raises the importance of considering HLH in this patient population. Prompt evaluation for underlying malignancies and infectious etiologies in patients with HLH, particularly those with atypical presentations, allowed for the initiation of appropriate treatment and ultimately led to a favorable outcome for our patient. Further research is needed to better understand the pathophysiology and optimal management strategies for patients with HLH and associated malignancies.
